# Two‐Phase Epoxidations with Micellar Catalysts: Insights, Limitations, and Perspectives

**DOI:** 10.1002/cplu.202500122

**Published:** 2025-04-16

**Authors:** Markus Hegelmann, Mirza Cokoja

**Affiliations:** ^1^ Department of Chemistry and Catalysis Research Center School of Natural Sciences Technical University of Munich Ernst‐Otto‐Fischer Straße 1 D‐85748 Garching bei München Germany

**Keywords:** additives, biphasic catalysis, epoxidation, ionic liquids, stimuli‐responsiveness

## Abstract

Biphasic molecular catalysis is a promising strategy for combining catalyst recycling with the synthesis of advanced chemical products. The anchoring of catalysts to surfactants in water allows for both catalyst solubility in aqueous media and a simple separation from the organic product. In biphasic epoxidations, this approach allows the use of environmentally benign hydrogen peroxide as oxidant. However, challenges remain due to mass transport limitations between the aqueous and organic phase, incompatibilities in the multicomponent system, and side reactions in the acidic medium. Hence, the development of surface‐active catalysts that enable controlled phase separation from all other components is highlighted in this concept article.

## Graphical abstract

Biphasic phase‐transfer catalysis is a promising strategy for catalyst recycling, which has successfully been applied to various industrial‐scale reactions. Its extension to oxidation reactions is, however, challenging. This concept article discusses epoxidations in water using surface‐active catalysts and emphasizes potential pathways toward more sustainable catalytic systems by using tailor‐made, multitasking stimuli‐responsive surfactants.

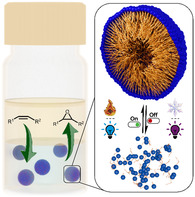

## 1 Introduction

Most industrial chemical processes rely on heterogeneous catalysts due to their advantages in catalyst reuse and recycling.^[^ ^1^ ^]^ However, the harsh reaction conditions often constrain these processes to simple bulk chemicals. In contrast, the strengths of molecular transition‐metal catalysts in homogeneous phase are typically manifested in the production of fine chemicals and pharmaceuticals.^[^ ^2^ ^]^ The intricacy of separating high‐performance molecular catalysts from the products in solution is a problem from the point of view of both sustainability and a broader applicability.^[^ ^3^ ^]^ To address the growing need for sophisticated value‐added products while minimizing environmental impact, more sustainable production methods are essential. Catalytic systems that operate in or on water are widely used for various organic transformations, offering the key advantage of easy product separation.^[^ ^4^, ^5^, ^6^ ^]^ This strategy has been successfully applied in industrial hydroformylations,^[^ ^7^, ^8^ ^]^ C–C cross couplings,^[^ ^9^, ^10^ ^]^ and hydrogenation reactions.^[^ ^11^, ^12^ ^]^ These reactions require late transition metal catalysts containing ligands such as triphenylphosphine trisulfonate, which impart high water solubility allowing for their use in biphasic aqueous‐organic systems. This design enables efficient catalyst separation and recycling and simple extraction of the pure organic product after the reaction, while quasihomogeneous conditions ensure high selectivity and efficiency in producing complex chemical products.^[^ ^13^, ^14^ ^]^ To further enhance the catalytic performance in these systems, surface‐active reagents were employed to solubilize the organic substrates in the aqueous phase, offering a pathway to sustainable large‐scale operations.^[^ ^15^, ^16^, ^17^ ^]^ This approach enables efficient catalyst recycling and the effective synthesis of advanced chemical products.^[^ ^18^, ^19^, ^20^, ^21^, ^22^, ^23^ ^]^ However, applying this concept to (ep)oxidation catalysis in water is difficult due to the inherent complexities and incompatibilities of the reaction components and compartments. While classic heterogeneous epoxidation catalysts only work with ethylene and propylene,^[^ ^24^ ^]^ virtually all other olefins are converted to epoxides by molecular catalysts in homogeneous phase, which are separated from the product either by distillation, or the fate of the catalyst is not relevant.^[^ ^25^ ^]^ Given the high importance of epoxides as intermediates in the synthesis of all kinds of fine chemicals, concepts for epoxidations of hydrophobic olefins in water, especially using hydrogen peroxide as oxidant, are most certainly warranted, yet underdeveloped.

## 2 The Recycling Challenge of Molecular Epoxidation Catalysts

Typically, olefin epoxidations in homogeneous phase are not designed to recover the catalyst, which undermines sustainability principles, particularly when (expensive) transition metal catalysts are involved. A notable example is the Sharpless epoxidation, which uses Ti‐based catalysts for producing chiral epoxides.^[^ ^26^ ^]^ This method is limited to allylic alcohols and requires strictly anhydrous, temperature‐controlled conditions along with unsustainable oxidants like *tert*‐butyl hydroperoxide.^[^ ^27^ ^]^ Another case is the asymmetric Jacobsen epoxidation with chiral manganese salen complexes.^[^ ^28^, ^29^ ^]^ Low reaction temperatures (<5 °C) and hazardous oxidants like iodosobenzene and bleach (owing to the incompatibility of the salen ligand with aq. H_2_O_2_) render this system impractical for large‐scale applications.^[^ ^30^ ^]^ Efforts to adopt the environmentally friendly H_2_O_2_ as oxidant have led to developments of noncatalytic Prilezhaev epoxidations.^[^ ^31^ ^]^ While this approach broadens the substrate scope, it suffers from drawbacks such as the stoichiometric use of acetic or formic acid, which generates large quantities of waste or leads to acid‐catalyzed epoxide ring opening.^[^ ^32^ ^]^ Biphasic epoxidation of hydrophobic nonpolar olefins therefore emerges as a promising solution, leveraging aqueous‐organic systems to enhance sustainability, catalyst recycling, and product selectivity.

## 3 Biphasic Epoxidation Catalysis

### 3.1 Liquid–Liquid Epoxidations

**Figure 1:**
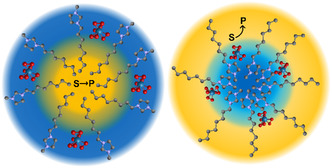
Illustration of the two general concepts of biphasic liquid–liquid epoxidation catalysis with an imidazolium cation as surfactant and a metal‐peroxo catalyst in aqueous media. Left: Solubilization of the organic substrate (yellow) in the catalyst‐containing aqueous phase (blue). Right: Transfer of the catalyst to the organic phase (quasihomogeneous conditions).

To enable efficient catalysis under biphasic conditions, either the catalyst must be transferred from the aqueous phase to the organic phase containing the hydrophobic substrate or vice versa (**Figure** **1**). The most refined approach to mediate between these immiscible phases is the use of micellar phase transfer agents.^[^ ^18^ ^]^ These surface‐active compounds form micelles when their concentration exceeds a specific threshold known as the critical micelle concentration (CMC). The formed supramolecular aggregates are stabilized by weak intermolecular forces, making them highly sensitive to external factors like temperature, additives, or solvent composition.^[^ ^33^, ^34^, ^35^ ^]^ Note that the CMC can be further tuned by increasing or decreasing the detergent properties, e.g., by introduction of longer alkyl chain which significantly lower the CMC.^[^ ^20^, ^22^ ^]^ Furthermore, the surfactant of choice has to meet other criteria: it has to be stable toward oxidative conditions, it must not decompose H_2_O_2_, and it must be exclusively soluble in water for product separation without catalyst loss. Classic ionic surfactants, such as cetyltrimethylammonium bromide, are soluble in organic media to a certain extent and therefore they are not viable for catalyst separation and reusability. Besides, halide anions decompose H_2_O_2_ at elevated temperatures (halide oxidation). Other common surfactants do not solubilize hydrocarbons in aq. H_2_O_2_ or contain functional groups which affect the epoxide selectivity, especially in acidic media (which is the case in aq. H_2_O_2_).

Polyoxometalates (POMs) are the most prominent catalysts for biphasic epoxidations since the pioneering studies by Venturello and Ishii.^[^ ^36^, ^37^ ^]^ POMs are readily available and cheap; however, their anionic oxo cluster structure, paired with simple alkali counterions, makes them solely water‐soluble. To enable epoxidation in biphasic systems, the active peroxo‐POM species must be transferred to the organic substrate phase by an external cationic phase‐transfer catalyst (PTC), such as tetraalkylammonium salts.^[^ ^19^, ^20^ ^]^ This results in very high catalytic activities, but recycling efforts with POM‐based systems are rare, since in most cases the catalyst remains in the product phase, which hinders upscaling toward sustainable applications outside of the laboratory scale.^[^ ^38^, ^39^, ^40^ ^]^

### 3.2 Catalysts for Epoxidations in Water

**Figure 2:**
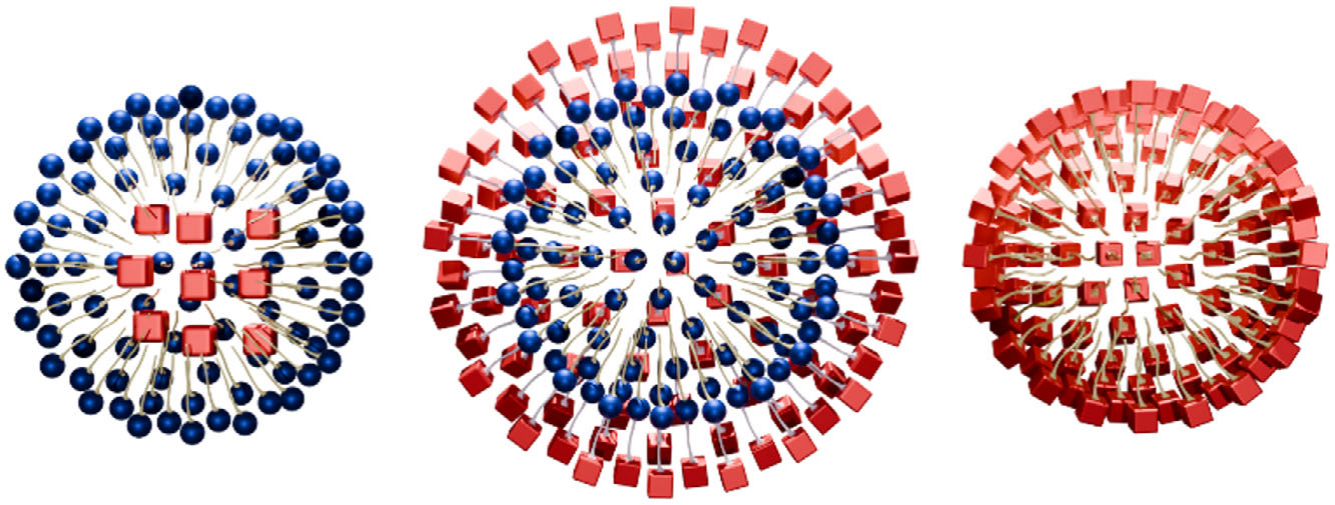
Schematic representation of cross sections of micellar aggregates, illustrating three different strategies for incorporating catalytically active species (red cubes) within surfactants (blue spheres) that form micellar structures. Left: The catalyst in encapsulated inside of the micelles of an external surfactant. Middle: The catalyst is covalently bound to the surfactant. Right: One‐component system where the surfactant is the catalyst itself.

For catalysis in water using a molecular catalyst and a surfactant, there are three general types of surfactant‐catalyst interactions to keep the catalyst in water, as illustrated in **Figure** **2**: encapsulation of the catalyst within the micelles (left),^[^ ^41^ ^]^ a chemical bonding of the catalyst to the surfactant (middle),^[^ ^42^ ^]^ and the catalyst itself being the surfactant (right).^[^ ^43^, ^44^, ^45^, ^46^, ^47^ ^]^

In contrast to POM systems, the approach of designing solely water‐soluble (one‐component) surfactants enables organic substrates to be transferred and solubilized in the aqueous oxidant phase.^[^ ^43^, ^44^, ^45^, ^46^ ^]^ This is generally advantageous, as it ensures the separation of the catalyst from the product, requiring only the catalyst to be isolated from the aqueous phase for recycling purposes. Note that some surfactants can also act as emulsifying agents, leading to the formation of stable emulsions between immiscible liquids, which subsequently require additional separation techniques.^[^ ^48^ ^]^

Imidazolium‐based ionic liquids (ILs) are stable and particularly versatile surfactants, offering a wide range of structural permutations that allow for fine‐tuning properties suited for applications in academia and industry, as electrolytes,^[^ ^49^, ^50^ ^]^ reaction media or additives in (biphasic) reactions,^[^ ^51^, ^52^, ^53^ ^]^ and as surface‐active (epoxidation) catalysts.^[^ ^20^, ^54^ ^]^ For epoxidations, surface‐active ILs (SAILs) can act as inert PTCs to solubilize organic compounds in water—and vice versa—by addition of a catalytically active metal salt^[^ ^55^, ^56^, ^57^ ^]^ or they can be designed as one‐component catalysts when paired with catalytically active counterions. Examples of catalytically active one‐component systems include SAILs containing tungstate,^[^ ^44^, ^46^, ^47^ ^]^ nitrate,^[^ ^45^ ^]^ or perrhenate anions.^[^ ^43^, ^47^ ^]^ The former activates H_2_O_2_ via an inner‐sphere mechanism to form a peroxo species, which is more efficient in converting olefins, compared to the outer‐sphere activation of H_2_O_2_ by [ReO_4_]^−^ and [NO_3_]^−^ anions.^[^ ^44^ ^]^ Hydrophobic perrhenate SAILs, for instance, dissolve and are surface‐active in aqueous H_2_O_2_ and allow catalyst recovery via precipitation after H_2_O_2_ consumption as they are immiscible with water.^[^ ^43^ ^]^ To ensure efficient and selective oxidation, the catalysts must be compatible with the respective oxidant in these systems, without undergoing decomposition or unwanted side reactions. This necessitates the absence of halides and trace metals, which can catalyze undesired H_2_O_2_ degradation. The alkylimidazolium‐based SAILs with catalytically active element oxoanions are immiscible with nonpolar organic media, such as most unfunctionalized olefins, the corresponding epoxides, and hydrocarbon solvents. They form micelles in aq. H_2_O_2_, and subsequently solubilize and convert the organic substrate therein, which principally allows product separation and catalyst recycling. A notable exception is polar substrates, such as allylic alcohols, which exhibit solubility in water (as do the epoxidation products), so that the systems are homogeneous. The concept of micellar catalysis with SAILs has been successfully applied to scale epoxidation reactions in continuous reactors;^[^ ^57^ ^]^ however, a sustainable process is yet to be developed due to the persisting problems biphasic epoxidations in water. In this regard, such reactions intrinsically suffer from: 1) incompatibility of the products with the acidic aq. media leading to acid‐mediated epoxide ring opening; 2) accumulation of water: the side product leads to breaking up of the aggregates below their CMC and catalyst deactivation; 3) separation of the catalyst from the aq. phase often limits quantitative recovery of the catalyst and recycling purposes require energy intensive distillation; and 4) phase separation: the increased polarity of the epoxide induces surfactant‐like properties hindering the full separation of the reaction compartments.

## 4 Advancing Biphasic Epoxidations with Stimuli‐Responsive Surface‐Active Catalysts?

**Figure 3:**
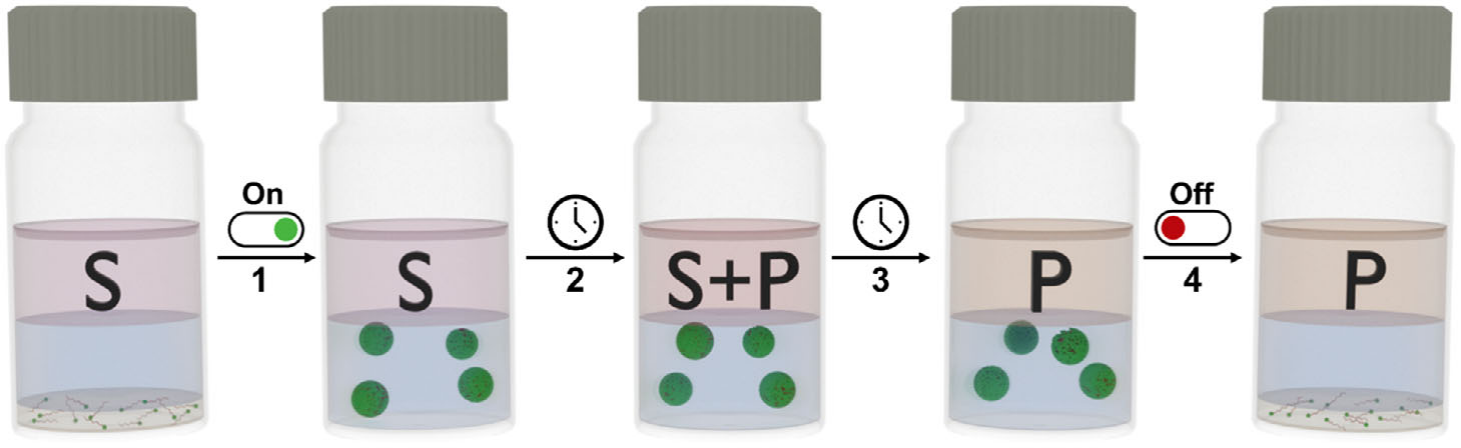
Concept of biphasic olefin epoxidation with stimuli‐responsive one‐component SAIL catalysts. Upon exposure to an external stimulus, the catalyst dissolves in the aqueous phase and forms micelles (step 1). The micellar catalyst solubilizes the substrate (S) in the aqueous phase and converts it to the epoxide product P (step 2). After full conversion (step 3) a reverse stimulus is applied, which induces the disassembly of the micelles and precipitation of the catalyst (step 4). This allows full catalyst separation from both the product and from water.

To tackle the challenges associated with state‐of‐the‐art surface‐active catalysts, they can be functionalized with stimuli‐responsive moieties that trigger the aggregation/disaggregation of micellar structures. This approach enables precise control over phase behavior in response to external stimuli such as temperature, light, redox reactions, magnetism, and pH variations.^[^ ^58^, ^59^, ^60^ ^]^ Reports on stimuli‐responsive surfactants show promising concepts, with these smart materials being applied in material science^[^ ^61^ ^]^ and in the biotechnological field for over a decade.^[^ ^62^ ^]^ However, very few reports deal with the transfer of these responsive systems toward catalytic applications. The development of catalytically active stimuli‐responsive surfactants remains in its early stages, posing a highly interesting research field for both academia and industry. These catalysts offer significant advantages, including a precise control over catalytic activity and simplified product separation and catalyst recycling, as illustrated in **Figure** **3**. Therefore, molecular epoxidation catalysts should be designed toward controllable phase behavior, which requires the introduction of specific functionalities to the surface‐active catalyst that enable responsivity to external stimuli. Note that not all of the functionalities that induce responsivity are suitable for biphasic (ep)oxidation reactions, primarily due to the used oxidant H_2_O_2_, which is environmentally benign and therefore should not be replaced by hazardous substitutes that potentially need additional separation techniques to remove them from the product. In this regard, pH‐responsive materials, which are mostly applied as drug delivery systems,^[^ ^63^, ^64^ ^]^ offer interesting properties for catalytic applications such as the tunability of the surface‐active properties and therefore the CMC at distinct pH values.^[^ ^65^ ^]^ However, pH‐sensitive catalysts are generally not suitable for biphasic epoxidations due to the generally acidic environment in aqueous H_2_O_2_.

Similarly, redox‐responsive surfactants deliver promising results in biochemical procedures^[^ ^66^ ^]^ and can also be adapted into catalytically active surfactants via anion exchange of ferrocenium (Fc)‐based ammonium salts, such as ferrocenyldimethylundecylammonium bromide. Upon oxidation of these Fc‐based surfactants, the respective CMC increased by several factors due to the increased solubility of the oxidized and highly charged species.^[^ ^67^, ^68^ ^]^ While these catalysts would allow for a switchable control over the micellization and the catalytic performance, in case of epoxidation reactions the used oxidant H_2_O_2_ is catalytically decomposed by the Fe^2+^‐containing compounds and is therefore not applicable.

### 4.1 Temperature‐ and Reaction‐Controlled Surfactants

**Figure 4:**
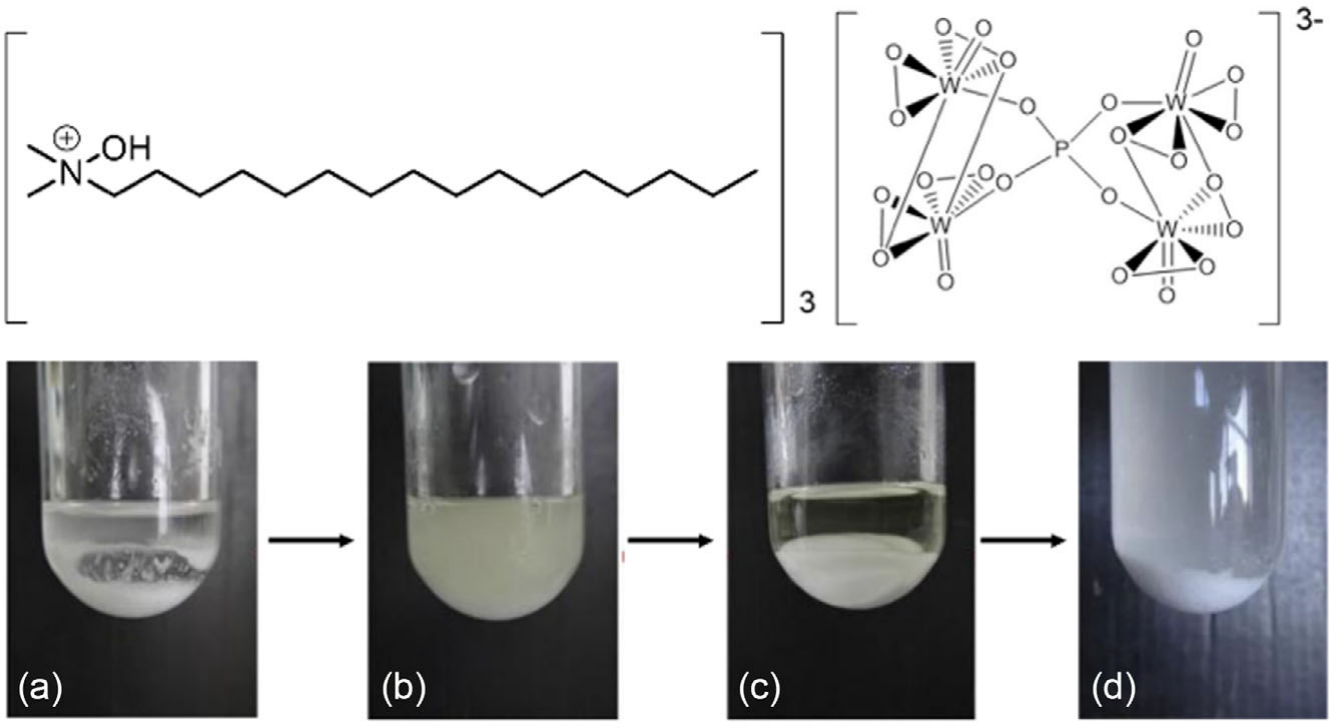
Top: Structure of the reaction‐controlled POM catalyst. Bottom: Biphasic epoxidation of cyclooctene with Wang's catalyst and H_2_O_2_: a) before reaction; b) during the reaction; c) at the end of reaction; d) at the end of the reaction after adding an excess of acetonitrile. Adapted with permission.^[^ ^75^ ^]^ John Wiley & Sons, Inc./CC BY 4.0.

Thermoresponsive materials are well studied in the field of polymer science, with extensive research focused on tailoring physicochemical parameters to develop materials with specific upper or lower critical solution temperatures (UCST, LCST) at which the solubility and miscibility with aqueous media change drastically.^[^ ^69^ ^]^ This concept was also expanded toward the design of molecular SAILs with distinct UCSTs and LCSTs, which were successfully applied as recyclable catalysts for various organic reactions.^[^ ^70^ ^]^ In multiphase systems, these thermoresponsive SAILs enable quasihomogeneous reactions under specific temperature conditions to efficiently convert the organic substrates, while phase separation into three distinct layers occurs upon heating above the LCST or cooling below the UCST.^[^ ^70^ ^]^ The research field was further expanded with examples of temperature‐controlled biphasic (ep)oxidations using functionalized amphiphobic materials. Here, POMs with polyfluorinated quaternary ammonium cations were used as catalysts for the oxidation of cyclohexanol.^[^ ^71^ ^]^ Epoxidation of allyl chloride was reported with the nonfluorinated analogue [(C_18_H_37_)_2_(CH_3_)_2_ N]_3_{PO_4_[W(O)(O_2_)_2_]_4_},^[^ ^72^ ^]^ and the design of temperature‐responsive POMs was further extended toward the epoxidation of styrene^[^ ^73^ ^]^ and propylene.^[^ ^74^ ^]^ Recently, a similar, reaction‐controlled POM based on the structure [(C_16_H_37_)(CH_3_)_2_NOH]_3_{PO_4_[W(O)(O_2_)_2_]_4_, as shown in **Figure** **4** (top), was applied as epoxidation catalyst for cyclooctene.^[^ ^75^ ^]^ After the consumption of H_2_O_2_, this catalyst can be precipitated from the reaction by adding acetonitrile, which only partially recovers the catalyst. Amphiphobic fluorinated imidazolium tungstate SAILs show a temperature‐controlled solubilization and precipitation in aq. H_2_O_2_ and principally allow for a reversible temperature‐driven epoxidation.^[^ ^47^ ^]^ However, the formation of the epoxidation product and the additive phenylphosphonic acid (PPA) induces a quantitative phase transfer of the catalyst to the organic phase. This allows for a very efficient conversion of a variety of substrates, however, it hinders a quantitative catalyst recycling from the aq. phase via precipitation. In other cases, the formation of the epoxide leads to a reaction‐controlled phase separation, allowing for catalyst recycle and reuse.^[^ ^76^ ^]^ This showcases the importance of investigating the effect of dynamic changes of the catalytic setup, whether triggered by intrinsic parameters of the system or by external ones, such as additives. To date, a broad range of additives have been used to enhance the activity and selectivity of catalytic reactions.^[^ ^77^, ^78^ ^]^ A variety of additives ranging from organic bases to mineral and carboxylic acids are used to enhance the performance of tungsten‐based epoxidation catalysts.^[^ ^79^ ^]^ Since the seminal studies of Noyori, one of the most prominent examples is the use of (organo)phosphonic acid additives.^[^ ^80^ ^]^ However, the process of identifying the best additive to prevent catalyst transfer into the product phase relies heavily on trial‐and‐error studies.

For alkylimidazolium tungstate SAILs, the catalysis‐enhancing role of PPA was elucidated via mass spectrometry studies, revealing the formation of the catalytically active phosphonate‐di(peroxo) tungstate species, which remains in the aqueous phase before, during, and after the reaction.^[^ ^44^ ^]^ Note that PPA additionally acts as phase transfer agent only in the case of fluorinated SAILs, which are poorly soluble in water/H_2_O_2_.^[^ ^47^ ^]^ This underlines that additives, in this case PPA, which are primarily used to enhance catalytic system activity, often possess additional properties that can influence not only the conversion but also selectivity. For instance, they may affect selectivity by potentially generating oxidative byproducts, such as mineral acids. Furthermore, additional challenges can arise from the separation of the additive from the product, the catalyst, or both.

The studies shown in this section highlight the inherent limitations of temperature‐responsive catalysis systems. To develop an applicable and sustainable temperature‐controlled catalyst, it must meet the following criteria: 1) it should dissolve at the reaction temperature and exhibit both surface activity and catalytic functionality; 2) it must exhibit minimal solubility under ambient conditions (<0.1 mol%) to enable quantitative precipitation; and 3) it should not interact with additives or the organic substrate/product phase beyond its intended catalytic function.

The complexity of these requirements, along with the limited literature on such systems, underscores the intricate nature of phase‐transfer catalysis and temperature‐controlled catalysts. Nearly every component of the reaction mixture influences the catalyst's solubility, phase behavior, and reactivity, making it challenging to achieve a systematic and comprehensive understanding.

### 4.2 Light‐Responsive Surfactants

**Figure 5:**
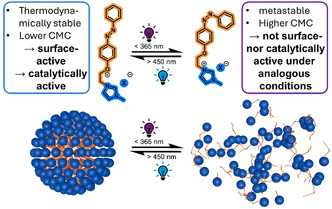
Top: reversible conversion of the thermodynamically stable *trans*‐form of an azobenzene‐functionalized SAIL to the *cis*‐form by UV‐light. Bottom: illustration of micellar aggregates formed by the surface‐active *trans*‐form and the disassembly to monomers upon photoisomerization.

Some of the challenges arising from intrinsic properties of temperature‐dependent systems can be addressed by employing catalysts, which undergo changes in the chemical structure upon application of other external stimuli. In this context, irradiation with light of different wavelengths and the subsequent changes in physicochemical parameters result in favorable properties such as switchable phase behavior due to polarity changes within the compound. SAILs offer a highly tunable structure motif for the incorporation of various functional groups. Chromophoric groups that respond to light in the UV–vis range are known for nearly a century.^[^ ^81^ ^]^ Compared to other stimuli‐responsive systems, this approach holds significant potential for designing sustainable biphasic (epoxidation) catalysts. While this material class is also relevant outside of the scope of catalysis,^[^ ^82^ ^]^ so far, there are only few reports on using light responsive surfactants for catalytic applications. The most prominent structure motifs of suitable chromophores for such systems include azobenzene^[^ ^83^ ^]^ and its heteroaryl derivatives,^[^ ^84^ ^]^ as well as diarylethene,^[^ ^85^ ^]^ and spiropyran.^[^ ^86^ ^]^ While all of these photoswitchable materials show different isomerization mechanisms that induce distinct properties, azobenzene‐based surfactants show the most promising reversible properties for applications in biphasic epoxidation catalysis, such as precise control over the assembly and disassembly of the micellar aggregates.^[^ ^87^ ^]^ Azobenzene derivatives are known to undergo full photoisomerization from the thermally stable *trans*‐isomer to the *cis*‐isomer upon irradiation with UV‐light (<365 nm), which is fully reversible upon heating or irradiation with visible light (>450 nm), with absorption ranges that can be specifically tailored.^[^ ^88^ ^]^ This photoswitch is highly efficient, yielding near‐quantitative photostationary states and inducing significant changes in molecular sizes, dipole as well as polarity.^[^ ^89^ ^]^ It is also noteworthy that azobenzene and its derivatives are stable to oxidative conditions, which is required for epoxidation catalysis.^[^ ^90^ ^]^ Previous studies have shown that upon light‐induced isomerization azobenzene‐containing surfactants either aggregate to form micelles, or they precipitate from water.^[^ ^91^, ^92^, ^93^ ^]^ In the context of recycling, such surfactants could be designed to 1) have one isomer soluble in the substrate phase (catalysis in the org. phase) and upon isomerization, the other isomer would undergo phase transfer into water, or 2) one isomer forms a third phase, and upon photoisomerization the surfactant forms micelles in water (catalysis in the aq. phase); a switch back to the initial isomer that disassembles and precipitates would allow for separation of the catalyst from water. This, in turn, could facilitate a continuous process using micellar catalysts by avoiding accumulation of water in the catalyst solution and an eventual micelle disassembly/catalyst deactivation. It has to be noted here that in the most cases both isomers of azobenzene‐containing surfactants are miscible with water. These transformations directly impact the solubility and phase behavior by changing the hydrophobicity/‐philicity. This is also reflected in direct changes in the CMC of the two photoisomers, in which the more stable *trans*‐form tends to have a lower CMC than the *cis*‐isomer.^[^ ^94^, ^95^, ^96^ ^]^ This property is particularly relevant for epoxidation catalysis, where the *trans*‐form remains surface‐active, effectively solubilizing organic substrates in water, while the *cis*‐form exists as monomers and is thus catalytically inactive (**Figure** **5**).

Note that there are only a handful of reports for the control over the aggregation and hence the activity/selectivity of light‐responsive micellar catalysts. In 2019, an amphiphilic system based on Zn^2+^‐complexed 1,4,7‐triaza‐cyclononane bearing a azobenzene moiety was investigated as a photoresponsive catalyst for the hydrolysis of esters.^[^ ^97^ ^]^ More recently, a chiral Rh catalyst was immobilized on an azobenzene‐containing photoresponsive surfactant and used in asymmetric transfer hydrogenations.^[^ ^98^ ^]^

The key requirement for a successful application of photoswitch concepts for micellar epoxidations lies in maintaining the catalyst concentration within a specific range (ΔCMC), which is above the CMC of the *trans*‐isomer but below that of the *cis*‐isomer, i.e., CMC_trans_ < ΔCMC < CMC_cis_. Existing photoresponsive surfactants, such as 4‐butylazobenzene‐4′‐(oxyethyl)trimethylammonium bromide and 4‐ethoxyazobenzene‐4′‐(oxyhexyl)trimethylammonium bromide, exhibit a CMC increase by factors of four and three, respectively, upon isomerization to the *cis*‐form.^[^ ^95^, ^96^ ^]^ However, their overall CMC values remain in the single‐digit mmol/L range, which is relatively low for potential SAIL‐based epoxidation catalysts. Consequently, for SAILs, two key factors should be considered: high water solubility to ensure effective dispersion and a sufficiently large difference between the CMC values of the two isomers to enable distinct phase behavior control. To achieve this, highly water‐soluble substituents, such as sulfonate groups, can be incorporated into the azobenzene moiety. Additionally, reducing the hydrophobic alkyl chain length and exchanging anions for more hydrophilic ones, such as nitrate, are potential strategies to enhance water solubility and, therefore, increase the CMC and the CMC gap between both photostationary states. Despite the inherent complexity and vast synthetic possibilities—leading to varied interactions with other substances—the initial studies have demonstrated the potential of these systems. By improving recyclability and sustainability in catalysis, photoresponsive SAILs present a promising pathway toward more controlled and efficient chemical processes within closed‐loop systems.

## 5 Summary and Outlook

We have shown the current concepts for two‐phase olefin epoxidations with surface‐active molecular catalysts in water, aiming at an efficient and long‐term recovery of the often expensive catalysts. While there are few studies highlighting epoxidations in water, they actually pinpoint the strengths and weaknesses of the state of the art. Most importantly, the solubilities of the catalyst in water and in the organic phase may well change over the course of the reaction, which depends on the polarity change of the medium, owing to the conversion of the olefin to an epoxide, which hinders the straightforward recovery and reuse of catalysts. The precise role and impacts of this dynamic interplay are still insufficiently studied. The lack of systematic understanding limits the deliberate tailoring of the catalyst to induce specific properties.

To address these challenges, future advancements in biphasic catalysis will focus on designing stimuli‐responsive catalysts. Incorporating external stimuli such as temperature or light can facilitate reversible phase separation and efficient catalyst recovery. This is already known for temperature‐controlled epoxidations, which showed promising results and feasible catalyst recycling to some extent. Among other stimuli‐responsive approaches, light‐driven catalysis stands out as the most promising. Unlike pH‐ or redox‐responsive systems, which are unsuitable in acidic and oxidative environments, photoresponsive catalysts, particularly those with azobenzene moieties, offer significant advantages. The design of such catalysts should aim to: 1) function as a single‐component catalytically active surfactant; 2) exhibit high solubility in aqueous media; 3) maintain stability in acidic and oxidative media; 4) achieve quantitative and reversible photostationary states; 5) display a substantial difference in CMC between photoisomers; and 6) undergo phase separation, ideally leading to catalyst precipitation, upon UV‐light irradiation.

Systematic investigations into the physicochemical properties of the (catalytically active) surfactants, additives, and dynamic reaction parameters are critical for unraveling mechanistic complexities, mitigating side reactions, and optimizing catalyst performance and reuse. Advancing these innovations could revolutionize the scalability of environmentally friendly catalytic processes, driving progress in green chemistry and enabling sustainable systems for industrial applications working in closed chemical cycles.

## Conflict of Interest

The authors declare no conflict of interest.
